# Items

**Published:** 1902-01

**Authors:** 


### ITEMS.

FRANK A. RUF, ESQ., president and treasurer of the Antikamnia Chemical Company, has just purchased a lot 80x109 feet, on the northwest corner of 22d and Pine Streets, St. Louis, for $20,000 cash, on which his Company will begin the erection, early in spring, of a new Antikamnia laboratory, five stories high, covering the entire lot. The improvements will cost about

$45,000 irrespective of the laboratory apparatus and appliances which will be of most approved pattern, from Darmstadt, Germany. The offices and various departments will be fitted with all modern conveniences, making the whole plant one of the most complete specialty laboratories in the United States. The success of the Antikamnia Company has been marvelous and the energy of its officers bespeaks a continuance of the enterprise on enlarged lines.

---

THE Rio Chemical Company, of New York (formerly of St. Louis) has published a handsome gallery of pictures of interest to physicians. They are reproductions in color of famous paintings—12 in number—and are entitled, "An Accident," "A Test," "Home Treatment," "A Ray of Hope," "A Hypnotic Experiment," "The Alchemist," "A Cure for Gout," "Lesson in Anatomy," "Dutch Doctor and Patient," "Convalescent," "An Old Doctor's Study," and "The Leech." They are artistic pictures and should be seen to be appreciated.

---

THE Arlington Chemical Company, of Yonkers, N.Y., has issued a portrait of William McKinley, late President of the United States, for gratuitous distribution. It is an enlarged reproduction in rich sepia tints, 17 x 13, of one of President McKinley's best photographs and is a remarkable faithful likeness, besides being a work of artistic merit. It is important to have it properly framed, and the publishers send a suggestion with each picture looking to that end. The picture will be sent on application to the above address.

---

THE M. J. Breitenbach Company, of New York, is furnishing to physicians the Year book for 1902, as in former years. It is one of the most useful desk memorandum books with which we are familiar. It is of convenient size, each page being devoted to a day, and upon each page is an appropriate reminder of some clinical use of pepto-mangan (Gude), which has taken a strong hold of the profession as a blood builder. The book will be sent on application to the publishers, 53 Warren Street, by any physician who has been overlooked in the distribution.

---

THE Mellier Drug Co., St. Louis, has issued a very artistic plaque on the reverse of which is given the story of tongaline, which is the popular name of a preparation composed of fluid extract of tonga, fluid extract of cimicifuga racemosa and the salicylates of sodium, pilcocarpin, and colchicin. It is indicated in neuralgia, rheumatism, grip, nervous headache, gout, malaria, and many diseases of the liver and kidneys. It is sold in liquid and tablet forms, and the tablets are offered plain or in combination with lithia and quinine. To prevent substitution it is advised that original packages be prescribed or only responsible druggists be patronised.

